# Quantitative proteomics and transcriptomics of potato in response to *Phytophthora infestans* in compatible and incompatible interactions

**DOI:** 10.1186/1471-2164-15-497

**Published:** 2014-06-19

**Authors:** Ashfaq Ali, Erik Alexandersson, Marianne Sandin, Svante Resjö, Marit Lenman, Pete Hedley, Fredrik Levander, Erik Andreasson

**Affiliations:** Department of Plant Protection Biology, Swedish University of Agricultural Sciences, Alnarp, Sweden; Department of Clinical Sciences, Lund University Diabetes Research Center, Malmö, Sweden; Department of Immunotechnology, Lund University, Lund, Sweden; The James Hutton Institute, Dundee, Scotland, UK

**Keywords:** Potato, Desiree, Sarpo Mira, SW93-1015, Secretome, Apoplast, Resistance, Hypersensitive response, *Phytophthora infestans*

## Abstract

**Background:**

In order to get global molecular understanding of one of the most important crop diseases worldwide, we investigated compatible and incompatible interactions between *Phytophthora infestans* and potato (*Solanum tuberosum*). We used the two most field-resistant potato clones under Swedish growing conditions, which have the greatest known local diversity of *P. infestans* populations, and a reference compatible cultivar.

**Results:**

Quantitative label-free proteomics of 51 apoplastic secretome samples (PXD000435) in combination with genome-wide transcript analysis by 42 microarrays (E-MTAB-1515) were used to capture changes in protein abundance and gene expression at 6, 24 and 72 hours after inoculation with *P. infestans.* To aid mass spectrometry analysis we generated cultivar-specific RNA-seq data (E-MTAB-1712), which increased peptide identifications by 17%. Components induced only during incompatible interactions, which are candidates for hypersensitive response initiation, include a Kunitz-like protease inhibitor, transcription factors and an RCR3-like protein. More secreted proteins had lower abundance in the compatible interaction compared to the incompatible interactions. Based on this observation and because the well-characterized effector-target C14 protease follows this pattern, we suggest 40 putative effector targets.

**Conclusions:**

In summary, over 17000 transcripts and 1000 secreted proteins changed in abundance in at least one time point, illustrating the dynamics of plant responses to a hemibiotroph. Half of the differentially abundant proteins showed a corresponding change at the transcript level. Many putative hypersensitive and effector-target proteins were single representatives of large gene families.

**Electronic supplementary material:**

The online version of this article (doi:10.1186/1471-2164-15-497) contains supplementary material, which is available to authorized users.

## Background

The oomycete *Phytophthora infestans*, the cause of potato (*Solanum tuberosum*) late blight disease, is one of the most confounding plant pathogens. Despite over a century of resistance breeding, fungicide use, and other control measures, it is still a major threat to sustainable food production worldwide. *P. infestans* is responsible for global annual costs of at least €5.6 billion in control measures and crop losses and is especially a threat to farmers’ income and food security in developing countries [1]. In addition, new regulations designed to reduce threats to the environment will limit the availability of fungicides, and conditions favoring overwintering spores due to the effects of global warming, are expected to increase problems with this disease [2].

*P. infestans* has a hemibiotrophic life cycle [3] and bi-phasic growth, with an initial biotrophic phase and subsequent necrotrophic phase of infection [3, 4]. The successful suppression of plant defense by effector molecules leads to a compatible interaction between plant and pathogen, which is referred to as effector-triggered susceptibility (ETS) [5]. A large number of extracellular and cytoplasmic effectors in the *P. infestans* genome have been identified [6, 7] and increasing evidence for their role in establishing ETS exists. However, knowledge about their targets in the plant host is scarce, mainly because of the limited availability of hemibiotrophic pathogens with susceptible interactions with the model species *Arabidopsis* [8]. Some of the *P. infestans* extracellular effectors are inhibitors that target defense-related proteins such as proteases and glucanases, and processes such as programmed cell death in plants. For example, Kazal-like extracellular serine protease inhibitors EPI1 and EPI10 inhibit the P69B subtilisin-like serine protease in tomato [9, 10], while others such as the cystatin-like protein target cysteine-proteases [11] and EPIC1 and EPIC2B inhibit papain-like cysteine protease RCR3, in the same plant [12]. These latter two inhibitors also target cysteine protease C14 by direct binding in the extracellular compartment [13]. In addition to extracellular inhibition of C14 by EPIC1 and EPIC2, an intracellular RXLR effector, AVRblb2, can prevent C14 secretion to the apoplast [14]. On the other hand, *P. infestans* has several xyloglucan-specific endoglucanases while the potato genome includes ten clustered genes for xyloglucans-specific endoglucanase inhibitor proteins (XEGIPs) [15].

Apart from PAMP (pathogen associated molecular patterns) recognition, plants have evolved a second mechanism of pathogen recognition through resistance genes, via direct or indirect interaction with effectors. Through this mechanism plants can avoid ETS, leading instead to an incompatible interaction between plant and pathogen, referred to as effector-triggered immunity (ETI). This is often accompanied by a hypersensitive response (HR) [5, 16]. PAMP and effector recognition leads to induction of biotic stress signaling involving MAP kinase cascades and induction of defense-related genes through phosphorylation of WRKY [17] and MYB transcription factors [18]. WRKY8 is involved in plant basal defense in tobacco, and is phosphorylated by MAPKs [19]. In addition, both jasmonic acid and salicylic acid are required for activation of PAMP-induced defense responses in potato [20].

Genome-wide transcript profiling has revealed many similarities between incompatible and compatible interactions, but higher numbers of differentially expressed genes were found during an incompatible *Hyaloperonospora*–*Arabidopsis* interaction [21]. However, genes with altered expression exclusively in compatible interactions have also been identified [21–23]. Microarrays based on cDNA clones [24–26], and more recently DeepSAGE transcriptome sequencing analysis, [27] have developed an understanding of compatible and incompatible interactions between *P. infestans* and potato, but none of these studies were based on the sequenced potato genome.

Here, we present a genome-wide expression profiling of compatible and incompatible potato–*P. infestans* interactions in combination with quantitative apoplastic secretome analyses. Although apoplastic profiling to find representative protein families has been performed in rice, chickpea, alfalfa and *Arabidopsis* unaffected by plant pathogens [28, 29], and in response to pathogens to a limited extent [30, 31], quantitative analysis of apoplast proteins during the progress of any compatible and incompatible interactions has not been presented previously. The recent release of the potato genome [32], combined with clone-specific sequence information determined by RNA-seq, increased sensitivity of current mass spectrometers, and new methods for label-free quantitative proteomic analysis enabled our study. These technological advances present new possibilities to help understand mechanisms in plant-pathogen interactions as well as to identify candidates for resistance against pathogens.

The Nordic countries have high *P. infestans* diversity, with both of the *P. infestans* mating types required for sexual reproduction and a suitable environment for sexual recombination [33]. Since these conditions provide an excellent environment to evaluate sustainable resistance against *P. infestans*, we have chosen the two most resistant clones based on several years of field trials in Sweden [34]. In addition, we monitor one compatible interaction, allowing us to find putative effector targets, many of which are single proteins from large gene families.

## Results

### Overview of differentially expressed genes and changes in protein abundance

In order to study stress responses in plant–oomycete interactions, we performed time series analyses of two incompatible interactions (Sarpo Mira and SW93-1015) and one compatible interaction (Desiree) with *P. infestans* (SE-03058). This was performed at a global level using genome-wide microarrays to determine gene expression changes and short 1D-gel separation followed by MS/MS analysis on a LC-coupled Orbitrap mass spectrometer to determine quantitative changes in apoplastic protein levels. Sarpo Mira and SW93-1015 have similar HR initiation, but later Sarpo Mira has a more typical R gene-mediated expansion in HR lesions [34]. For the compatible interaction, Desiree was chosen because it is the most used potato cultivar in molecular and physiological studies.

In our genome-wide array, 17451 transcripts were significantly differentially expressed relative to uninoculated control in at least one time point in one or more clones during the course of *P. infestans* challenge (Table 1). This demonstrates that this biotic stress condition has a profound effect on transcriptional responses in potato.

**Table 1:** **Number of differentially expressed transcripts in the microarray experiments in potato clones Desiree, Sarpo Mira and SW93-1015 and the number of proteins with differential abundance in the three potato clones at 6, 24 and 72 hpi**

Differentially expressed transcripts						
Potato clone	6 hpi		24 hpi		72 hpi	
	Up	Down	Up	Down	Up	Down
**Desiree**	1449	1602	633	618	6009	6109
**Sarpo Mira**	1362	1440	543	351	331	417
**SW93-1015**	1169	1131	1501	1782	2554	2989
**Proteins with differential abundance in the secretome**						
**Potato clone**	**6 hpi**		**24 hpi**		**72 hpi**	
	**Up**	**Down**	**Up**	**Down**	**Up**	**Down**
**Desiree**	218	76	194	82	289	225
**Sarpo Mira**	307	74	353	66	375	127
**SW93-1015**	273	45	397	41	307	38

**Figure 1:**
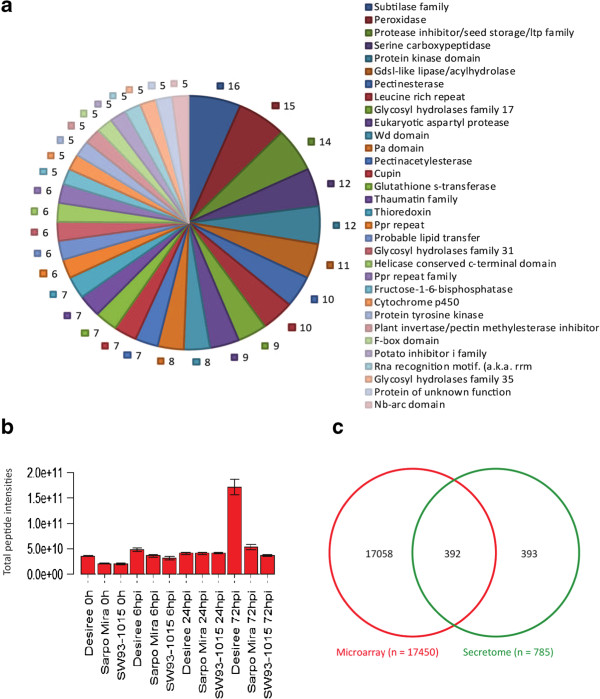
**Characterization of secreted proteins. (a)** Number of genes found in identified protein families in the apoplastic secretome based on conserved domains and motifs using InterProScan. **(b)** Mean total peptide intensities for each sample across the four replicates. Error bars indicate standard deviation of the mean. **(c)** Venn diagrams of differentially expressed transcripts in the microarray and differentially abundant proteins in the apoplast. Only the 785 proteins with Potato Genome Sequencing Consortium (PGSC) annotations from 1075 differentially abundant proteins were used for comparison.

In order to improve the peptide identification by mass spectrometry, we generated about 52 million clean pair-end reads from RNA-seq data for each of the three potato clones. Contigs were constructed by *de novo* transcriptome assembly and subsequently *ab initio* gene prediction algorithms were used to predict ORFs, which were then combined in a customized database for peptide identification. We found more than 3000 transcripts (ORFs) for each potato clone that lacked BLAST hits in the *S. tuberosum* predicted proteome (Additional file 1: Table S1) and an overall 17% increase in peptide identification was observed. From the apoplast of the three potato clones a total of 12591 peptides were identified. Peptides that appeared in at least two replicates per time point were kept, resulting in 5055 peptides corresponding to 1639 proteins, which were used for quantitative analysis. Of the 1639 identified proteins, 1075 were found to be differentially abundant in at least one time point during the infection, and of these 785 had Potato Genome Sequencing Consortium (PGSC) annotations. In total, 830 of the identified proteins could be classified into 433 different protein families by InterProScan analysis [34, 35]. Based on TargetP [36] analysis for secretory signals, 517 of these proteins were predicted to have a secretory signal, which is in line with other apoplastic secretome studies [29]. Among the most prominent protein families were subtilase family proteins, peroxidases, protease inhibitors, GDSL-lipases, and pectin esterases (Figure 1a). Several proteins that are classified as pathogenesis-related (PR) proteins were identified both in infected and un-infected samples in all three potato clones, including thaumatin, glucanases, glucosidases, P69 proteins, peroxidases, serine proteases, cysteine proteases, PRp27 protein, subtilases, chitinases, osmotins and lipid transfer proteins. Many PR protein family members are thus present in the apoplast already prior to infection and could be putative effector targets very early in plant–pathogen interactions.

At 6 hours post infection (hpi) and 24 hpi, similar total peptide intensity was observed in both compatible and incompatible interactions, suggesting similar amounts of protein at these time points. However, at 72 hpi we observed significantly higher total peptide intensity in the compatible interaction (Desiree) compared with the two resistant potato clones (Figure 1b) although fewer proteins with higher abundance were observed in Desiree (Table 1). At 72 hpi in Desiree, glucanases and glucosidases comprised a major part of the total apoplast protein intensity, whereas total peptide intensity for all identified proteases and peptidases increased in all three clones (Additional file 2: Figure S1). This indicates a more complex response of the protein composition in the incompatible interactions. For proteins that showed a change in abundance in the apoplast during infection only 392, or 50%, of the corresponding transcripts showed differential expression (Figure 1c).

**Figure 2:**
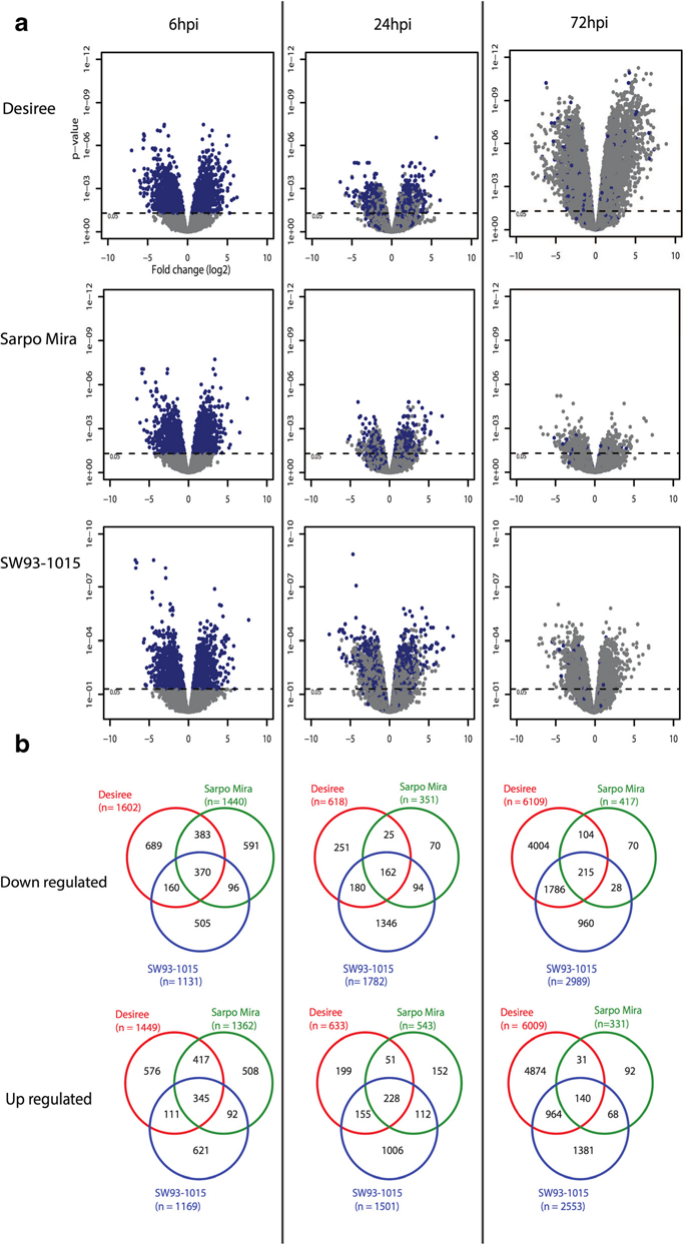
**Transcript analysis across the time course in Desiree, Sarpo Mira and SW93-1015. (a)** Volcano plots showing differentially expressed transcripts and overlap between the different time points. Blue dots at 24 and 72 hpi indicate transcripts with present at previous time points and grey dots indicate unique transcripts for that time point. **(b)** Venn diagrams of down-regulated transcripts (upper part) and up-regulated transcripts (lower part) regulated in the microarray experiment in the three potato clones at 6, 24, and 72 hpi from left to right.

A large number of transcripts were found to be differentially expressed at 6, 24, and 72 hpi in both compatible and incompatible interactions (Table 1). Different sets of transcripts were differentially expressed between earlier time points (6 and 24 hpi) and later time point (72 hpi) (Figure 2a), indicating a biphasic response to pathogen inoculation in both incompatible and compatible interactions.

Similar numbers of induced transcripts were observed in all three clones at 6 hpi (Figure 2b). However in Sarpo Mira fewer differentially expressed transcripts were observed compared with the other two potato clones at later time-points (Table 1, Figure 2). Uninfected SW93-1015 had around 4000 transcripts that were differentially expressed compared to un-infected Desiree controls. These included SA induced defense marker *PR1* gene family members (DMG400005109; DMG400005111), and JA induced defense marker [37] *MYC2* (DMG400017535). This supports our earlier conclusion that this clone has a paranoia trait, a weak constitutively active defense [34]. Furthermore, GO terms related to plant cell death and defense such as programmed cell death, methyl salicylate esterase activity, apoptotic process, and innate immune response were enriched in SW93-1015 control samples compared to Desiree control (Additional file 3: Table S2). SW93-1015 generally had more differentially expressed transcripts than Sarpo Mira at 24 and 72 hpi (Table 1), most of which were specific to SW93-1015 (Figure 2b). However, a large number of transcripts with altered expression in SW93-1015 were also differentially expressed in Desiree (Figure 2b), indicating that SW93-1015 might recognize more cues from *P. infestans* than Sarpo Mira.

**Figure 3:**
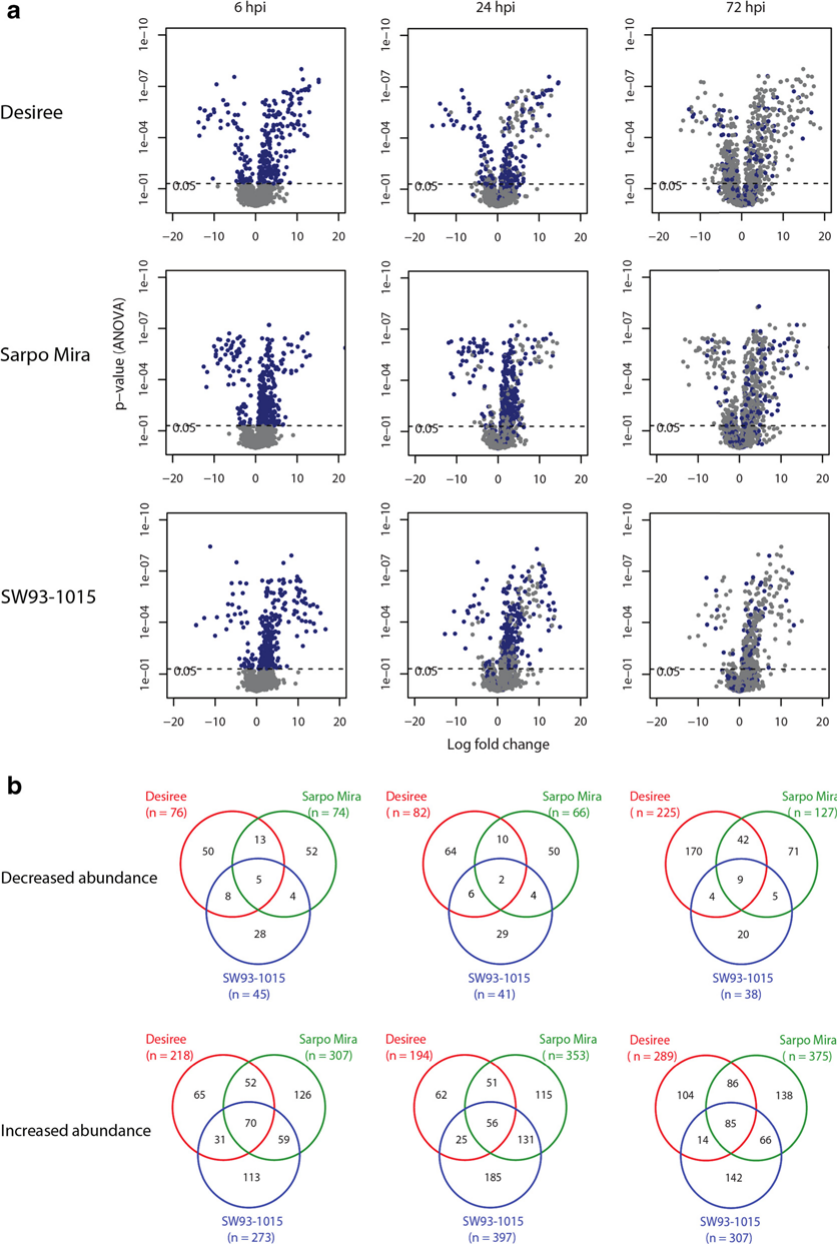
**Differentially abundant apoplastic proteins across the time course in Desiree, Sarpo Mira and SW93-1015. (a)** Volcano plots showing differentially abundant proteins. Blue dots at 24 and 72 hpi indicate proteins present at previous time points and grey dots indicate unique proteins for that time point. **(b)** Venn diagrams illustrating numbers of proteins with lower abundance (upper part) and higher abundance (lower part) in the apoplast of Desiree, Sarpo Mira and SW93-1015 from top to bottom at 6, 24, and 72 hpi from left to right.

Similar to the transcriptome patterns, several proteins that differed in abundance overlapped between 6 and 24 hpi within clones, as seen in the volcano plots (Figure 3a). At 72 hpi, however, several more proteins differentially accumulated compared with earlier time-points (Figure 2a). In addition, a larger number of proteins decreased in abundance in Desiree compared with Sarpo Mira and SW93-1015 (Figure 3a, Table 1). A corresponding difference in down-regulated transcripts was not observed. Instead, a much larger number of up-regulated transcripts was seen in Desiree at 72 hpi compared with the incompatible interactions, as mentioned above. A large number of proteins that increased in abundance were common in all three potato clones (Figure 3b). In addition, a higher percentage of proteins with increased abundance were observed during incompatible interactions in the apoplastic secretome data (Figure 3b) compared with transcript data where larger number of differentially expressed transcripts were observed in the compatible interaction (Table 1).

### Responses during incompatible interactions

**Figure 4:**
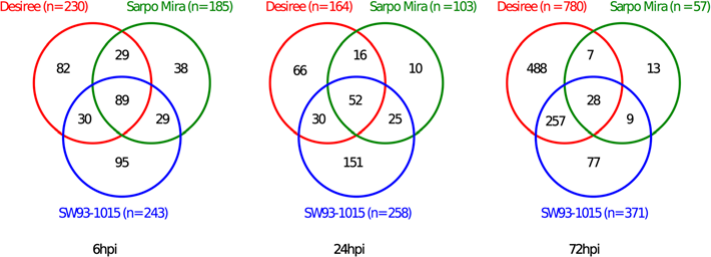
**Number of enriched GO terms based on the gene expression analyses of the three potato clones Desiree, Sarpo Mira and SW93-1015 at 6, 24, and 72 hpi.**

A hallmark for plant resistance towards pathogens is the initiation of the HR reaction, but in crops relatively little is known about the genes involved. Among the differentially expressed transcripts we found 92, 112, and 68 transcripts at 6, 24, and 72 hpi, respectively, that were up-regulated in both resistant potato clones but not in susceptible Desiree (Figure 2b). Some of these genes such as the MYB transcription factors, glutaredoxins, RING zinc finger proteins and U-box proteins, have members that have previously been associated with resistance (Additional file 4: Table S3). There were 29 different GO terms uniquely enriched for the early (6 hpi) incompatible interaction (Figure 4). Similarly we found 59, 131, and 66 secreted proteins at 6, 24, and 72 hpi, respectively, with increased abundance in the two incompatible interactions but not in the compatible interaction (Figure 3b). These include a subtilisin-like protease, Kunitz-like protease inhibitors, lipid transfer proteins, defensins and strictosidine synthase. These genes and proteins can be regarded as candidate involved in initiation of HR and resistance against *P. infestans*.

MapMan pathway analyses reveal that a majority of the represented MYB transcription factors are down-regulated at 6 hpi in Desiree, whereas the opposite is true for Sarpo Mira and SW93-1015 (Additional file 2: Figure S2). In contrast, several WRKY transcription factors are down-regulated in all three clones at 6 hpi.

In order to identify testable HR initiation candidates, we took a conservative approach and identified genes that were 4-fold up-regulated only in the resistant cultivars at 6 or 24 h, at the RNA (Table 2) and protein (Table 3) level. Only four of the 25 transcripts were differentially regulated in the apoplast (highlighted bold in Table 2). The 49 secreted proteins which accumulated early in resistant clones indicate that this compartment is active in HR initiation or other early resistance mechanisms (Table 3). Notable proteins from the apoplast are the RCR3-like Phytophthora-inhibited protease 1 (PIP1), the aspartic protease nepenthesin-1 and a Kunitz-like protease inhibitor. These findings show that the proteolytic machinery of potato plays an important role in defense against pathogens. In line with these findings, tomato plants with mutated *Rcr3*, which is related to PIP1, have enhanced susceptibility to *P. infestans* [12]. Several hydrolytic enzymes are also identified and these might exhibit novel catalytic specificities evolved to assist in ETI.

**Table 2:** **Candidates for hypersensitivity initiation from microarray data**

RNA transcripts			Increased in incompatible clones		
Gene ID	Protein ID	Name	6 h	24 h	72 h
PGSC0003DMG401010883	PGSC0003DMP400019223	R2r3-myb transcription factor	*		
PGSC0003DMG402004331	PGSC0003DMP400007701	Conserved gene of unknown function	*		
PGSC0003DMG400030212	PGSC0003DMP400052593	Nitrate reductase	*		
PGSC0003DMG400001855	**PGSC0003DMP400003321**	**Beta-amylase PCT-BMYI**	*		
PGSC0003DMG400036101	PGSC0003DMP400058205	Gene of unknown function	*		
PGSC0003DMG400019518	PGSC0003DMP400033920	Pseudo response regulator	*		
PGSC0003DMG400011502	PGSC0003DMP400020369	PEP carboxylase kinase	*		
PGSC0003DMG400020209	PGSC0003DMP400035084	Nodulin family protein	*		
PGSC0003DMG400001460	PGSC0003DMP400002648	Ninja-family protein Os03g0419100	*		
PGSC0003DMG400025240	PGSC0003DMP400043812	Axi 1 protein	*		
PGSC0003DMG400020498	PGSC0003DMP400035611	Inositol-1,4,5-triphosphate-5-phosphatase		*	
PGSC0003DMG400023702	PGSC0003DMP400041014	Calmodulin binding protein		*	
PGSC0003DMG400023949	PGSC0003DMP400041401	Abscisic acid receptor PYL4		*	
PGSC0003DMG400001771	PGSC0003DMP400003164	Dead box ATP-dependent RNA helicase		*	
PGSC0003DMG400001387	PGSC0003DMP400002507	Ocs element-binding factor		*	
PGSC0003DMG400010759	PGSC0003DMP400019038	Gene of unknown function		*	
PGSC0003DMG400000731	PGSC0003DMP400001424	Response to dessication RD2		*	
PGSC0003DMG400009982	PGSC0003DMP400017630	BTB/POZ domain-containing protein		*	
PGSC0003DMG400003993	PGSC0003DMP400007118	Citrate binding protein		*	
PGSC0003DMG400003057	**PGSC0003DMP400005490**	**Osmotin**		*	*
PGSC0003DMG400015267	**PGSC0003DMP400026776**	**Kunitz-type protease inhibitor**		*	*
PGSC0003DMG400026220	PGSC0003DMP400045511	Major pollen allergen Ory s 1		*	*
PGSC0003DMG400008100	PGSC0003DMP400014249	KiTH-2	*	*	*
PGSC0003DMG400008099	PGSC0003DMP400014248	KiTH-2	*	*	*
PGSC0003DMG400008098	**PGSC0003DMP400014247**	**KiTH-2**	*	*	*

Genes with increased expression in the two incompatible clones Sarpo Mira and SW93-1015, but not in compatible Desirée. Genes with a minimum 4-fold up-regulation in both Sarpo Mira and SW93-1015 at 6 or 24 hpi and stable or down-regulated in Desirée at 6 and 24 hpi were selected. Up-regulation is indicated with an asterisk. Bolded protein IDs and names indicate proteins for which abundance data was obtained. Symbol (*) indicates the time point in which the change was observed.

**Table 3:** **Candidates for hypersensitivity initiation from apoplast proteomics data**

Secreted proteins		Increased in incompatible clones		
Protein ID	Name	6 h	24 h	72 h
PGSC0003DMP400015021	Cellulase containing protein	*		
PGSC0003DMP400051976	Glucan endo-1,3-beta-D-glucosidase	*		
PGSC0003DMP400012140	Polyamine oxidase	*		
PGSC0003DMP400056168	Mitogen-activated protein kinase kinase kinase	*		
Q9LTJ3	Putative uncharacterized protein At5g59350	*		
Q9SWI1	Protein PHYTOCHROME KINASE SUBSTRATE 1	*		
DES_g15011_t1	Uncharacterized protein		*	
PGSC0003DMP400011041	Leucine-rich repeat family protein		*	
PGSC0003DMP400015631	Aldo-keto reductase family 4 member C10		*	
PGSC0003DMP400027722	Bacterial-induced peroxidase		*	
PGSC0003DMP400028029	Alpha-glucosidase		*	
PGSC0003DMP400030201	Strictosidine synthase		*	
PGSC0003DMP400046981	Kunitz trypsin inhibitor		*	
PGSC0003DMP400049292	Pattern formation protein		*	
PGSC0003DMP400051822	Actin-101		*	
PGSC0003DMP400001286 †	Conserved gene of unknown function †		*	
PGSC0003DMP400002450	Conserved gene of unknown function		*	
PGSC0003DMP400003176	Zinc finger family protein		*	
PGSC0003DMP400008097	Rho guanine dissociation inhibitor		*	
PGSC0003DMP400009086 †	PR1 protein †		*	
PGSC0003DMP400035459	Triacylglycerol lipase		*	
PGSC0003DMP400040683	ATP binding protein		*	
PGSC0003DMP400045856	Pentatricopeptide repeat-containing protein		*	
PGSC0003DMP400057833	Polyprotein protein		*	
Q9FJT0	Putative uncharacterized protein		*	
Q9LDP1	F28H19.2 protein (F2J6.15 protein)		*	
Q9LVB8	Probable carboxylesterase 120 (AtCXE20) (EC 3.1.1.1)		*	
Q9SCZ5	Putative uncharacterized protein F26O13.180		*	
PGSC0003DMP400005465	Osmotin	*	*	
PGSC0003DMP400018074	Phytophthora-inhibited protease 1	*	*	
PGSC0003DMP400021164	Non-specific lipid-transfer protein	*	*	
DES_g20722_t1	Uncharacterized protein	*	*	
O22214	Putative uncharacterized protein At2g41520	*	*	
O64572	Expressed protein (Uncharacterized protein)	*	*	
PGSC0003DMP400006538	Aspartic proteinase nepenthesin-1	*	*	
PGSC0003DMP400012829	Leucine-rich repeat receptor kinase	*	*	
PGSC0003DMP400026222	Cytochrome P450	*	*	
PGSC0003DMP400038388	Rhicadhesin receptor	*	*	
PGSC0003DMP400059150	Gene of unknown function	*	*	
PGSC0003DMP400067598	O-methyltransferase	*	*	
PGSC0003DMP400013560	Gene of unknown function		*	*
PGSC0003DMP400053911	Beta-galactosidase		*	*
PGSC0003DMP400012597	Epidermis-specific secreted glycoprotein EP1		*	*
PGSC0003DMP400016025	Pentatricopeptide repeat-containing protein		*	*
PGSC0003DMP400006168	Glycosyl hydrolase family	*	*	*
PGSC0003DMP400017287 †	Cytochrome P450 hydroxylase †	*	*	*
PGSC0003DMP400035498 †	Peroxidase †	*	*	*
PGSC0003DMP400064458	Gag-pol protein	*	*	*
Q9FKV2	Berberine bridge enzyme (FAD-binding and BBE domain-containing protein)	*	*	*

Proteins with increased abundance in the secretome for the two incompatible clones Sarpo Mira and SW93-1015, but not for compatible Desirée. Proteins with a minimum 4-fold increase in both Sarpo Mira and SW93-1015 at 6 or 24 hpi and stable or decreased abundance in Desirée at 6 and 24 hpi were selected. Up-regulation is indicated with an asterisk. The symbol † indicates proteins that were not identified using at least one peptide specific for the listed gene. Symbol (*) indicates the time point in which the change was observed.

### General defense and the susceptible interaction

There was a clear overlap between differentially expressed transcripts in both the incompatible and compatible interactions (Figure 2b) demonstrating that there are common sets of genes differentially expressed independently of the type of interaction. At 6 hpi there is a large overlap between all three clones in enriched GO terms (Figure 4) with many GO terms linked to primary metabolism, but also general signaling mechanisms such as protein tyrosine and serine/threonine kinase activity. A change in primary metabolism and brassinosteroid-related processes is then seen throughout the time course in all three clones (Additional file 3: Table S2). Between the three clones at 6 hpi, the MapMan pathway analysis shows a striking similarity for C2H2-CO-like and C2H2-Dof zinc finger containing transcription factors indicating that these might be involved in general defense (Additional file 2: Figure S1). There are a large number of GO terms only enriched in the compatible interaction (Figure 4). In hormone-related processes, auxin transport and stimulus GO terms are unique to the compatible interaction, and this is also true for gibberellin biosynthesis and stimulus. At 72 hpi a large number of GO terms are significantly enriched in Desiree. It is the only time point where functional groups associated with jasmonic acid and systemic acquired resistance are present, and is in line with the start of the necrotrophic phase of the pathogen. Also, several histone-related genes can be seen to be up-regulated in Desiree, whereas this is the case for only a few members in SW93-1015 and none in Sarpo Mira.

### Screen for putative effector targets in the apoplast

**Figure 5:**
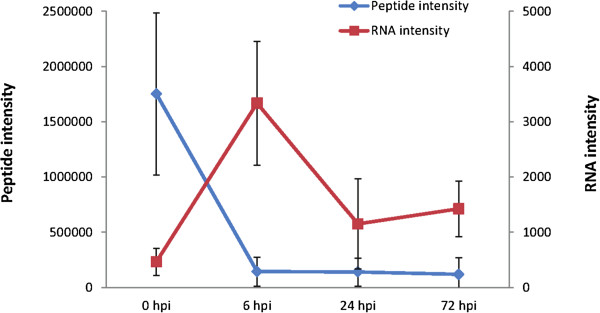
**Gene expression and protein abundance in the apoplast of known effector target cysteine protease C14.** Transcript and protein levels of the effector target cysteine protease C14 decreased at 6 hpi in the Desiree apoplast and at the same time was transitionally up regulated.

Although the use of effector targets in resistance breeding is desirable [38], very few of such proteins have been identified in oomycete–plant interactions. We detected many secreted proteins that decreased in abundances during the compatible interaction. This is in line with a suppression of some secreted proteins by pathogen effectors or a faster degradation due to protein-complex formation. *P. infestans* effector can suppress secretion of a defense protein is exemplified by the cysteine protease C14 [14]. We found that the C14 protease decreased sharply at 6 hpi in the Desiree apoplast even though it increased at transcript level at this time point validating Bozkurt et al’s findings in tomato (Figure 5). This inspired two strategies to screen for putative effector targets in our quantitative apoplastic secretome data.

Firstly, we selected proteins with a minimum of 8-fold decreased in abundance in the susceptible clone at 6 and 24 h after infection. This selection produced 40 candidates, many of which are single proteins from large gene families (Table 4). These proteins may also be putative targets in the incompatible interactions because at early time points secreted effectors are expected to be present in all interactions.

**Figure 6:**
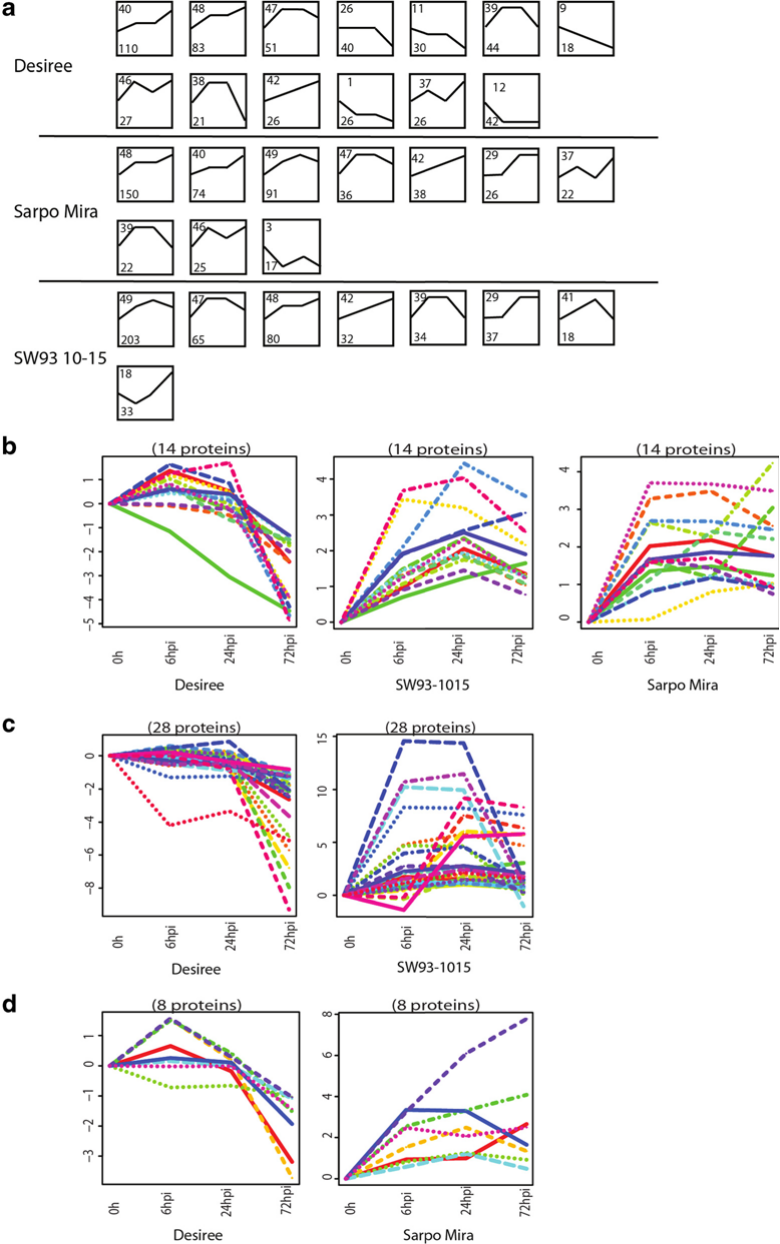
**Identification of candidates for effector targets. (a)** Significant profiles from STEM clustering of apoplastic proteins at 0, 6, 24, and 72 hpi in the three potato clones. Protein intensities from four replicates for each sample were used for clustering. Only proteins with statistically significant differences for at least at one time point were used. Each box represents a protein abundance pattern during the time course. Top left of each box is the profile number defined in STEM and bottom left of each box indicates the number of proteins that fit the defined profile pattern. **(b)** Proteins found with decreased abundance profiles in Desiree, but increased abundance profiles in both Sarpo Mira and SW93-1015. **(c)** Proteins found with decreased abundance profiles in Desiree but with increased abundance profiles in SW93-1015. **(d)** Proteins found with decreased abundance profiles in Desiree but with increased abundance profiles in Sarpo Mira.

Secondly, we performed clustering by STEM, in order to find protein abundance patterns that occur during compatible and incompatible interactions in the apoplast. Among 13 profiles with significant numbers of associated proteins in Desiree, we found five profiles (number 26, 11, 9, 1, and 12 in order of significance) containing in total 156 proteins with lower abundance in the apoplast. In contrast, only one significant profile in Sarpo Mira (out of 10 profiles) and none in SW93-1015 (out of 11 profiles) represented proteins with lower abundance (Figure 6a). In addition, one of the profiles representing proteins with higher abundance (49), was only significant for SW93-1015 and Sarpo Mira, and represented a large number of proteins for these clones, 203 and 91 proteins respectively (Figure 6a).

**Table 4:** **Putative effector targets**

Protein ID	Name	Desiree 6 h	Desiree 24 h
PGSC0003DMP400017664	Transcription factor	*	
Q9SZ87	Reverse transcriptase (RNA-dependent DNA polymerase)	*	
Q9SPM5	GDA1/CD39 (nucleoside phosphatase) family	*	
Q8LBK6	Glutaredoxin	*	
Q9SSD1	Leucine rich repeat	*	
PGSC0003DMP400018076	Cysteine protease	*	
PGSC0003DMP400045977	Cystatin	*	
PGSC0003DMP400024264	Conserved gene of unknown function	*	
PGSC0003DMP400020961	5′-nucleotidase sure	*	
O81459	ATPase family associated with various cellular activities (AAA)	*	*
PGSC0003DMP400049952	Pectinesterase-2	*	*
F4J1D9	MutS domain II	*	*
PGSC0003DMP400008301	Pto-interacting protein 1	*	*
Q8VZM7	Protein of unknown function (DUF1012)	*	*
Q9FJV2	FBD, Leucine Rich Repeat	*	*
PGSC0003DMP400006604	Beta-galactosidase †	*	*
PGSC0003DMP400021388	HSP transcription factor †	*	*
PGSC0003DMP400001052	Peptidyl-prolyl cis-trans isomerase	*	*
PGSC0003DMP400054971	Phospholipase A1 †	*	*
PGSC0003DMP400065569	Gene of unknown function	*	*
DES.g31837.t1		*	*
PGSC0003DMP400056405	Histidine kinase 3B	*	*
PGSC0003DMP400016823	Kunitz-type proteinase inhibitor	*	*
PGSC0003DMP400044843	Serine-threonine protein kinase, plant-type	*	*
PGSC0003DMP400004370	Glutamate decarboxylase 4a	*	*
Q9FGS4	Fe-S metabolism associated domain, Quinolinate synthetase A protein	*	*
A0ME24	Putative uncharacterized protein	*	*
O81861	Glycosyl hydrolases family 18	*	*
PGSC0003DMP400008705	Subtilase family protein	*	*
PGSC0003DMP400068385	Conserved gene of unknown function	*	*
PGSC0003DMP400004622	Beta-galactosidase	*	*
PGSC0003DMP400055439	Protein kinase atmrk1	*	*
PGSC0003DMP400017510	Cysteine protease 14	*	*
PGSC0003DMP400017719	Multicopper oxidase		*
Q8VZG2	Domain associated at C-terminal with AAA, ATPase family associated with various cellular activities (AAA)		*
PGSC0003DMP400048051	Glutathione S-transferase T5		*
Q4FE22	Potato inhibitor I family		*
PGSC0003DMP400049086	Thioredoxin peroxidase		*
Q9FMR7	Mitochondrial fission ELM1		*
PGSC0003DMP400025698	High mobility group protein		*

Proteins with decreased abundance in the secretome for the compatible clone Desiree, but not for the two incompatible clones Sarpo Mira and SW93-1015. Proteins with a minimum 8-fold down-regulation in Desiree and stable in both Sarpo Mira and SW93-1015 at 6 or 24 hpi were selected. 8-fold down-regulation is indicated with an asterisk. The symbol † indicates proteins that were not identified using at least one peptide specific for the listed gene. Symbol (*) indicates the time point in which the change was observed.

In order to find putative effector-targeted proteins that are differentially regulated in compatible and incompatible interactions, we selected candidates from profiles containing proteins with increased levels in the two incompatible interactions (profile 39, 40, 42, 47, 48, and 49 from Sarpo Mira, and profile 29, 39, 41, 42, 47, 48, and 49 from SW93-1015) and compared them with the proteins associated with profiles representing decreased levels in the compatible interaction (profile 1, 9, 11, 12, 26) (Figure 6a). We found 14 proteins from both incompatible interaction profiles with increased abundance that showed decreased accumulation in the compatible interaction (Figure 6b; Additional file 5: Table S4). These included a lyase, a pectinesterase, a Kunitz trypsin inhibitor and a dehydratase. In addition, we found 28 proteins with decreased abundance profiles in Desiree (Profile 1, 9, 11, 12, 26) but with increased abundance profiles in SW93-1015 (Figure 6c; Additional file 5: Table S4). Eight proteins with decreased abundance profiles in Desiree were found in the increased abundance profiles of Sarpo Mira (profile 39, 40, 42, 47, 48, and 49; Figure 6d; Additional file 5: Table S4).

## Discussion

We have carried out time course studies of one compatible and two incompatible interactions between *P. infestans* and potato by global transcriptomics and apoplastic proteomics using potato clones Desiree, Sarpo Mira, and SW93-1015. In response to *P. infestans* infection, there was a large overlap of transcripts and proteins with changed abundance irrespective of compatibility, which can be seen as a common signature for challenge with *Phytophthora* (Figures 2b and 3b). Among the differentially abundant proteins found in the apoplast, 50% were also differentially expressed at the transcript level (Figure 1c). This is in line with previously reported data from yeast that showed that regulation of mRNA explains around 40% of the protein concentration changes [39], and demonstrates the value of measuring both transcript and protein levels.

We identified more than 1500 proteins in the apoplast; almost half of these are associated with 400 conserved functional domains (Figure 1a, Additional file 4: Table S3). The wide range of functional categories among apoplastic secretome suggests a high complexity in this protein fraction. Among the identified proteins 30% had a secretory signal predicted by TargetP, which is consistent with other apoplastic proteomics studies and suggests that a large proportion of the secreted proteins are secreted through unconventional secretory pathways [40].

At 6 hpi, a higher number of apoplast proteins increased in both incompatible interactions than in the compatible interaction, demonstrating a quicker response during the incompatible interaction (Table 1). This difference is even clearer at 24 hpi, when the number of proteins with increased abundance in the incompatible interactions is almost twice that of the compatible interaction. Another notable difference in the apoplast at this stage is that more proteins decreased in abundance in the compatible interaction compared to the incompatible interactions. Interestingly, we did not observe this difference in the transcriptome, indicating a more pronounced suppression by effectors on secreted proteins than on transcription. At 72 hpi, substantially more genes were induced in the compatible interaction than in the incompatible interactions. Most of these genes may have a role in metabolic and structural re-modeling due to infection.

Although the total protein content is greater in the compatible interaction (Figure 1b), fewer proteins show increased abundance. This suggests that the composition of the apoplastic secretome is important in the incompatible interaction and that certain apoplastic proteins are suppressed by the pathogen during the compatible interaction (Figure 1b; Tables 2 and 3). Major proteins that accumulated in the susceptible interaction were glucanases and glucosidases (Additional file 2: Figure S1a) that might be involved in symptom development, as many more cells were visibly affected in the compatible interaction than in the incompatible interactions [34]. Evidence that the apoplastic secretome is altered during pathogen attack has previously been found in cell cultures [41], but such a large-scale perturbation of the host apoplastic secretome *in planta* is unprecedented.

Little is known about the genes involved in development of HR in potato. There were few genes that were uniquely induced in both resistant potato clones; notably glutaredoxins, MYB transcription factors and a zinc finger protein (Tables 2 and 3; Additional file 4: Table S3). Plants with impaired glutaredoxin activity are resistant to the necrotroph *Botrytis* [42] and SA-inducible glutaredoxin is involved in suppression of the jasmonic acid-responsive genes in *Arabidopsis* [43], suggesting a role in hormone crosstalk during the defense response. Plant U-box proteins, also unique to incompatible interactions, is a gene family with diverse functions linked to salicylic acid and PAD4 mediated pathways [44]. WRKY transcription factors did not show this pattern and many were initially down-regulated, although WRKY8 has been implicated in defense-related MAPK signaling and *P. infestans* resistance in *Nicotiana benthamiana* [19].

Sarpo Mira, which carries five different resistance genes [45], showed relatively few changes after challenge with *P. infestans*. This might be due to specific R gene recognition and responses to *P. infestans*. Although the difference between specific responses in the two resistant genotypes was large, both still display full resistance against the pathogen and initiate similar macroscopic HR in detached leaf assays [34]. Our analysis suggests the possibility that pathogen recognition occurs differently in Sarpo Mira and SW93-1015. Sarpo Mira might mainly depend on effector recognition by resistance proteins, while SW93-1015 may have additional recognition and response mechanisms. At 6 hpi in SW93-1015 two LRR receptor-like kinases are induced, perhaps leading to defense responses unique to SW93-1015.

The fact that certain proteins in the apoplast might be selectively suppressed during the compatible interaction but not the incompatible interactions allows us to screen for putative effector targets. In this unique type of screening described here, we found several candidates that were single proteins from large gene families. In addition to the well characterized cysteine protease 14 (PGSC0003DMP400017510), we found another cysteine protease (PGSC0003DMP400045977) as well as a cystatin homologue (a putative cysteine protease inhibitor PGSC0003DMP400018076). Two other proteins of interest were a Kunitz-type proteinase inhibitor (PGSC0003DMP400016823) and a subtilase family protein (PGSC0003DMP400008705) that also was identified in this screen. With the exact identity of these genes it is now possible to investigate these functionally.

## Conclusion

We carried out a time course study of potato transcriptomic and proteomic responses after *P. infestans* inoculation, both in compatible and incompatible interactions. Studying samples from the same time course for both gene expression and protein abundance lead to identification of potential targets for plant proteins directly manipulated by the pathogen. By using two phenotypically different resistant potato clones we identify several transcripts and proteins that only show increased expression or abundance in both incompatible potato clones that are potentially involved in resistance. In summary, we provide ample number of transcripts and proteins that could be used in targeted studies on one of the most agriculturally important plant-pathogen interactions.

## Methods

### Plant material and growth conditions

Three potato clones Desiree, Sarpo Mira and SW93-1015 were used. Sarpo Mira and SW93-1015 are two highly resistant potato clones with slightly different resistance reactions [34]. Sarpo Mira is a potato cultivar that recognizes five different effectors from *P. infestans* [45] and shows a classical HR reaction in response to *P. infestans* inoculation. SW93-1015 is a breeding potato clone which is consistently resistant to Swedish *P. infestans* populations with reduced HR expansion and a weak *cpr* genotype [34]. Plants were grown in a growth chamber with controlled conditions set at 20°C with a 16:8 light:dark cycle and 70% relative humidity. Five-week-old plants were transferred to an infection chamber with 100% humidity and 10:14 light:dark cycle. After 6 hours, plants were sprayed with an encysted zoospore suspension from *P. infestans* isolate SE-03058 [46] until the leaf surfaces were fully saturated with the zoospore suspension (15000 sporangia/ml). Relative humidity was maintained at 100% for 2 days after inoculation and then adjusted to 90% for the rest of the experiment. Samples were collected at 6, 24, and 72 hpi. For RNA and apoplastic secretome sampling, fully-expanded upper leaves were collected.

### RNA isolation and microarray analysis

Samples were collected from three independent biological experiments. Four to six leaves frozen in liquid nitrogen were homogenized to a fine powder using FastPrep-24 (MP-bio, Santa Ana, USA) with 2 mm beads. Leaf tissue (100 mg) was weighed out and put into RNase-free tubes before extraction. RNA extractions were performed using the RNeasy Plant Mini kit (Qiagen GmbH, Hilden, Germany). Samples were DNase treated and cleaned using the Qiagen RNA cleanup kit. RNA concentration and purity (260/280 nm > 1.8) was checked by a ND-1000 NanoDrop (Wilmington, USA) and integrity of the samples were analyzed with an Experion™ Automated Electrophoresis System (Bio-Rad Laboratories, Hercules, USA). For mRNA expression analysis, a custom-made Agilent expression array (JHI *Solanum tuberosum* 60 k v1; ArrayExpress accession A-MEXP-2272) based on predicted transcripts of the *Solanum tuberosum* genome (version 3.4) was used according to the supplier’s directions (One-Color Microarray-Based Gene Expression Analysis Low Input Quick Amp Labeling v. 6.5; Agilent). Data were extracted from each scanned array image using Feature Extraction software (v. 10.7.3; Agilent). The array data is deposited in ArrayExpress: E-MTAB-1515.

### Apoplastic secretome sample preparation

Three biological replicates of un-infected control samples were collected. For each apoplast sample, four fully expanded middle leaves from two plants were subjected to independent apoplast isolations. In order to collect un-infected control samples, plants were transferred to the humid chamber and kept in the chamber for 6 hours under the same experimental conditions as for infection experiments. Apoplast isolation was performed by using vacuum infiltration as described [34]. Apoplastic fluids collected from two plants for each sample were pooled, dissolved in 6× SDS-PAGE buffer containing DTT, and denatured at 95°C for 3 minutes. Pooled samples (30 μl of each) were loaded on polyacrylamide gels and separated for 2 cm with SDS-PAGE. After staining with Coomassie, the gel lane from each sample was cut into about 1 mm^2^ pieces. Each lane was kept separate from this point on, generating five subreplicates for each control sample and four subreplicates for each infected sample. Samples were then subjected to in-gel tryptic digestion with incubation (modified sequencing grade; Promega, Madison, WI, USA) overnight at 37°C. Peptides were extracted in 50–80% acetonitrile and excess acetonitrile was vaporized using centrifugation under vacuum. De-salting was performed using UltraMicro spin columns (Nest group). The whole experiment was repeated twice.

### Mass spectrometry

MS analysis was performed on a LTQ Orbitrap XL with an Eksigent nano-LC system (Eksigent technologies, Dublin, CA, USA). A 5 μl sample was injected and separated at a flow rate of 300 nl/min with a 90 minute gradient. The four most intense ions were selected in data-dependent mode for fragmentation in the linear ion trap, for details see [47]. Files were converted to mzML [48] and Mascot Generic Format (MGF) using ProteoWizard [49] and uploaded to the Proteios Software Environment [50]. MGF files were used for MS/MS identification, and mzML files for feature detection. Identification searches were performed in Mascot (http://www.matrixscience.com) and X!Tandem (http://www.thegpm.org/tandem) in a database consisting of all *Solanum* proteins in UniProt (http://www.uniprot.org), all annotated proteins from the potato genome project (http://www.potatogenome.net, [6]) and predicted proteins from *de novo* assembled transcripts from three potato clones Desiree, Sarpo Mira and SW93-1015, plus an equally sized decoy part consisting of the reversed protein sequences (in total 670584 proteins). The MS mass tolerance was set to 5 ppm and MS/MS fragment tolerance to 0.5 Da, with one missed cleavage allowed. Cysteine carbamidomethylation was set as fixed and methionine oxidation as variable modification. FDR was subsequently calculated for the combined search results at the peptide-spectrum level, and filtered at a FDR of 1% as described previously [50, 51]. To quantify possible peptides, msInspect [52] feature detection was performed on the mzML files from Proteios using default settings. The features were matched to identifications with a retention time tolerance of 0.2 minutes and an *m/z* tolerance of 0.005 Da as well as a requirement of same charge and LC-MS/MS run. To facilitate the differential expression analysis and to propagate sequences between the runs, alignment was performed using our recently developed algorithm within Proteios [47, 53]. A report of the peptide features corresponding between runs was exported for further analysis. Peptide data used for further analysis is found in Additional file 6: Table S5 and LC-MS runs are listed in supporting information, Additional file 7: Table S6. The mass spectrometry proteomics data have been deposited to the ProteomeXchange Consortium (http://proteomecentral.proteomexchange.org) via the PRIDE partner repository with the dataset identifier PXD000435 and DOI 10.6019/PXD000435.

### Bioinformatics and statistical analysis

#### RNA-sequencing

We obtained approximately 52 million reads for each potato clone from RNA samples collected at 24 hpi using paired-end libraries from Illumina HiSeq 2000 machines. Clean paired-end reads were then *de novo* assembled using Trinity (version r2011-11-26) to build transcript contigs. Protein coding sequences from these contigs were extracted by gene prediction program Augustus (version 2.5.5) that was used for constructing a proteomics database. BLASTX was used to obtain annotations from the potato genome. The RNA-seq data has been deposited in ArrayExpress: E-MTAB-1712.

#### Microarray

Probe intensities were background-corrected and normalized using the quantile method in the Limma R package [54]. Genes with p-values below 0.05 after adjustment with the Benjamini-Hochberg method were regarded as significant. The projected GO annotation (Additional file 8: Table S7) [55] for the differentially expressed probes was then analyzed for enrichment of Gene Ontology (GO) terms using GOEast [56] with default settings (adjusted p-value < 0.1).

#### Apoplastic secretome

Peptides with a FDR of < 0.01 were selected for further analysis. For normalization, we used the Eigen MS method incorporated in DanteR (v0.2) that uses Eigen values to find trends in the data for normalization. Only peptides that were identified in at least two of the subreplicates of any sample were selected and normalized. In DanteR, data was filtered, missing values imputed and protein level intensities for the leading proteins calculated using Q-Rollup function with 2 to 3 peptides per protein based on median peptide intensities [57–59]. Proteins with single peptide hits were included for further analysis. Differentially expressed proteins were calculated using one-way ANOVA for all time points from each potato clone with their relative controls. Fold change estimates were performed based on linear model comparison of each time point with the relative control for each potato clone. After Benjamini-Hochberg (BH) p-value adjustment, differentially expressed proteins with adjusted p-value < 0.05 were selected for further analysis. Default settings in STEM [60] were used and. Standalone InterProScan [35] with default settings was used to find conserved domains and peptide fingerprints for apoplastic secretome protein classification [61]. TargetP was used for secretory signal peptide identification [36]. MapMan analysis was performed to explore gene pathways based on sequence identity to *Arabidopsis* genes [61].

## Electronic supplementary material

Additional file 1: Table S1: Clone-specific peptide identifications. ORFs were predicted based on RNA-seq data in Desirée, Sarpo Mira and SW93-1015. (PDF 9 KB)

Additional file 4: Table S3: Gene expression during *Phytophthora* infection. Microarray data was normalized and analyzed with the Limma R-package. Expression ratios and corresponding adjusted p-values are given for all microarray probes with matching potato transcripts. (ZIP 11 MB)

Additional file 3: Table S2: List of enriched Gene Ontology terms across the time course. Enrichment was done with GOEast using default settings and a cut-off of p < 0.1. (TXT 118 KB)

Additional file 2: Figure S1: Total peptide intensity for proteases and peptidases. a, Sum of total intensities of 49 proteins in the apoplast identified as glucanases and glucosidases (4 replicates) b, Sum of total intensities of 78 proteins in the apoplast identified as proteases and carboxypeptidases (4 replicates). Error bars indicated ± SD of the mean. **Figure S2.** Functional analysis of gene expression by MapMan. Bins for genes related to hormones **(a)** and transcription **(b)** for Desirée, Sarpo Mira and SW-1015 at 6, 24 or 72 hpi with an adjusted p-value of <0.05 are shown. (PDF 1 MB)

Additional file 5: Table S4: Protein identifiers for resistance candidates identified in STEM profiles. Excel file with identifiers correspond to profiles presented in Figure 6b-d. (PDF 55 KB)

Additional file 6: Table S5: Peptide data during *Phytophthora* infection. Raw mass spectrometer peptide intensities. (TXT 4 MB)

Additional file 7: Table S6: LC-MS file names. Raw data was have been deposited to the ProteomeXchange Consortium (http://proteomecentral.proteomexchange.org) via the PRIDE partner repository with the dataset identifier PXD000435 and DOI 10.6019/PXD000435. (PDF 32 KB)

Additional file 8: Table S7: Gene ontology terms for the potato genome and custom-made Agilent expression array. The file (Extended_GOterms_JHI_Solanum_tuberosum_60kv1_expression_array.txt) is formatted for use in GOEast [57]. (TXT 4 MB)
