# Supplementary material for: Quantitative proteomics and transcriptomics of potato in response to Phytophthora infestans in compatible and incompatible interactions
Source: BMC Genomics. 2014 Jun 19;15(1):497. doi: 10.1186/1471-2164-15-497 (PMC4079953; doi:10.1186/1471-2164-15-497)
Supplement: Supplementary file 1 — Additional file 1: Table S1: Clone-specific peptide identifications. ORFs were predicted based on RNA-seq data in Desirée, Sarpo Mira and SW93-1015. (PDF 9 KB) [file 12864_2014_6185_MOESM1_ESM.pdf]

Table S1: Predicted ORFs based on RNA-seq data in Desirée, Sarpo Mira and SW93-1015.

**Cultivar**	**Number of contigs**	**Number of transcripts predicted by Trinity**	**No significant blast hits in Phureja proteome**	**Number of peptides identfied (Phureja)**	**Peptides (Phureja+ Uniprot)**	**Unique Peptides (Phureja + RNA seq)**
Desiree	97794	36925	3569	8675	9036	10432
Sarpo Mira	96153	36353	3521			
SW93-1015	91370	35059	3544			
