# Supplementary material for: Quantitative proteomics and transcriptomics of potato in response to Phytophthora infestans in compatible and incompatible interactions
Source: BMC Genomics. 2014 Jun 19;15(1):497. doi: 10.1186/1471-2164-15-497 (PMC4079953; doi:10.1186/1471-2164-15-497)
Supplement: Supplementary file 4 — Additional file 2: Figure S1: Total peptide intensity for proteases and peptidases. a, Sum of total intensities of 49 proteins in the apoplast identified as glucanases and glucosidases (4 replicates) b, Sum of total intensities of 78 proteins in the apoplast identified as proteases and carboxypeptidases (4 replicates). Error bars indicated ± SD of the mean. Figure S2. Functional analysis of gene expression by MapMan. Bins for genes related to hormones (a) and transcription (b) for Desirée, Sarpo Mira and SW-1015 at 6, 24 or 72 hpi with an adjusted p-value of <0.05 are shown. (PDF 1 MB) [file 12864_2014_6185_MOESM4_ESM.pdf]

Supporting Information Figure S1: **Total peptide intensity for proteases and peptidases**. **a,** Sum of total intensities of 49 proteins in the secretome identified as glucanases and glucosidases (4 replicates) **b,** Sum of total intensities of 78 proteins in the secretome identified as Proteses and carboxypeptidases (4 replicates). Error bars indicated ±SD of the mean.

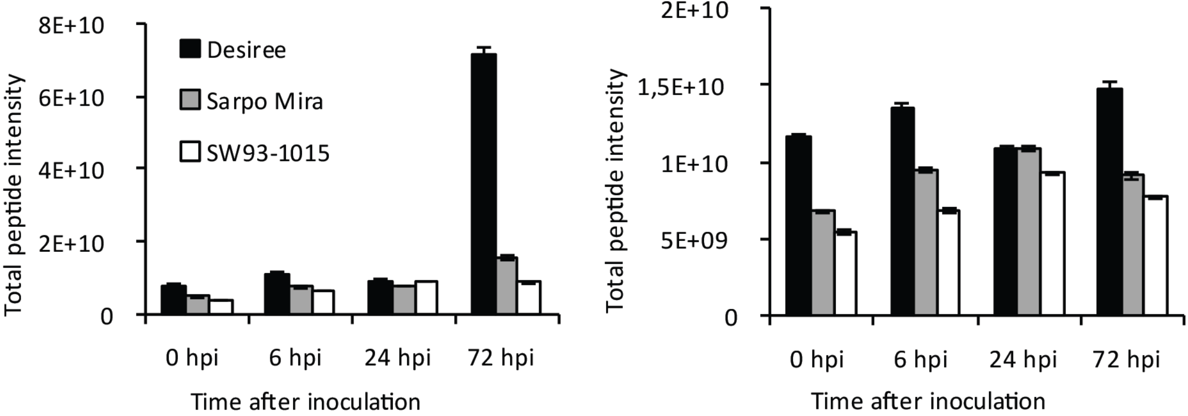

8E+10
6E+10
4E+10
2E+10
0
Total peptide intensity
Desiree
Sarpo Mira
SW93-1015
0 hpi
6 hpi
24 hpi
72 hpi
Time after inoculation
2E+10
1,5E+10
1E+10
5E+09
0
Total peptide intensity
0 hpi
6 hpi
24 hpi
72 hpi
Time after inoculation

Supporting Information Figure S2: **Functional analysis of gene expression by MapMan.** Bins for genes related to hormones (a) and transcription (b) for Desirée, Sarpo Mira and SW-1015 at 6, 24 or 72 hpi with an adjusted p-value of <0.05 are shown.

Figure S2a

Desiree – transciption factors

6 h

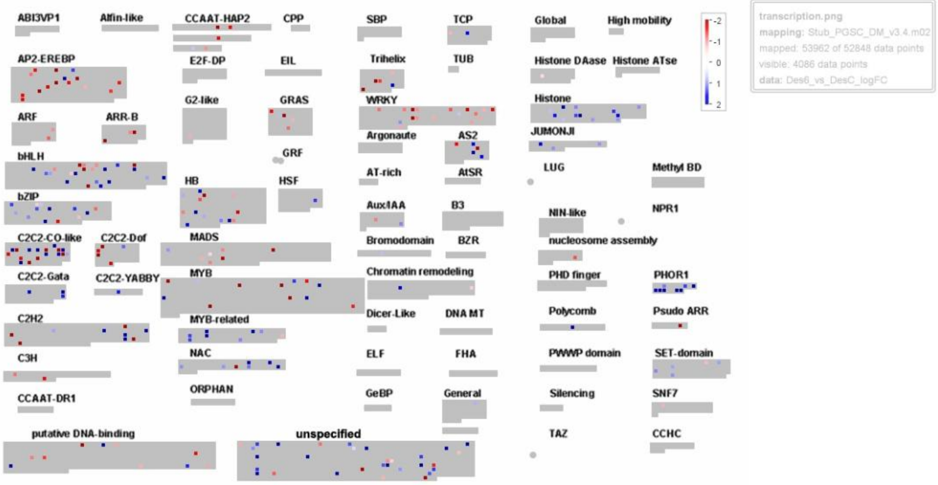

24 h

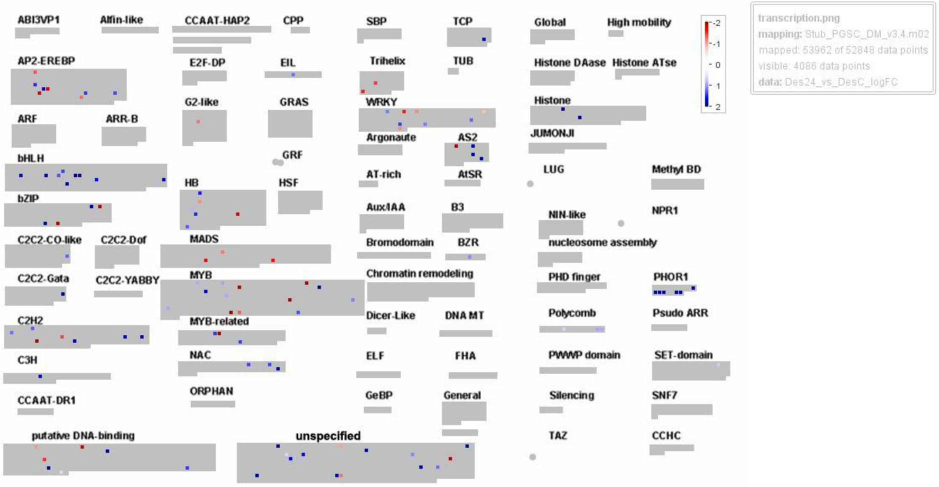

72 h

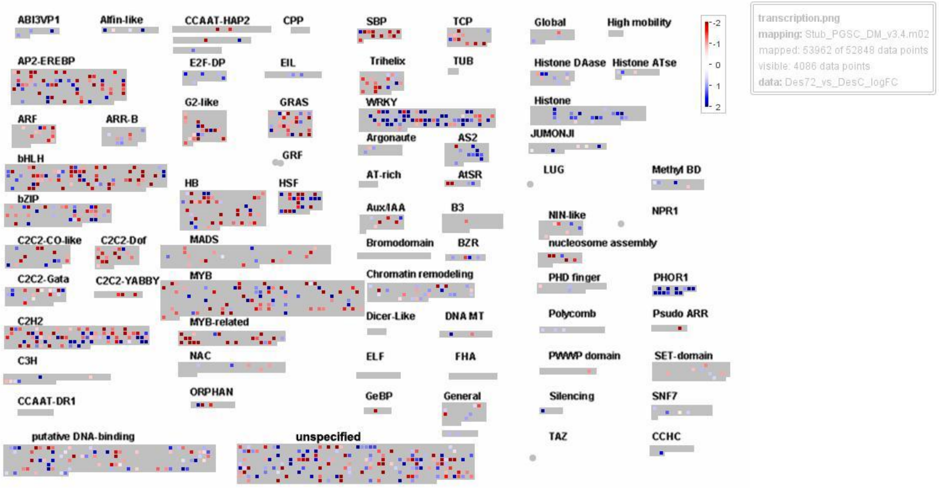

Sarpo Mira – transciption factors

6 h

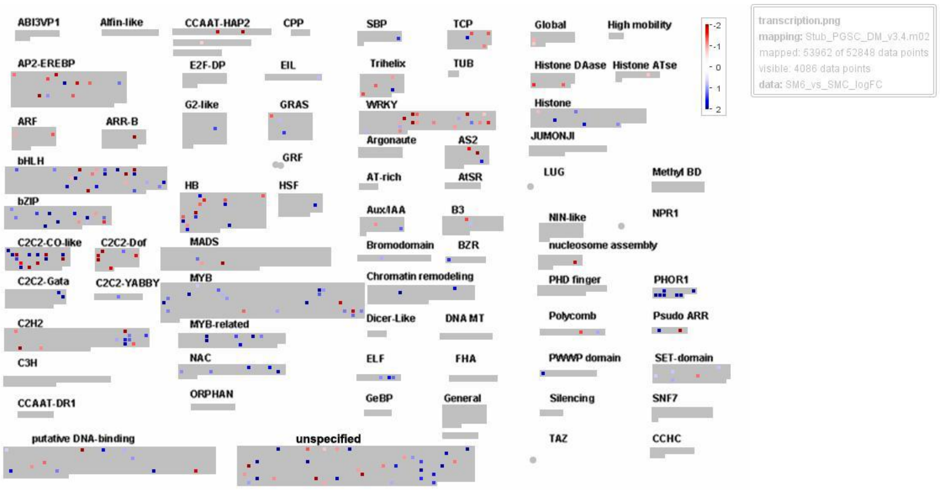

24 h

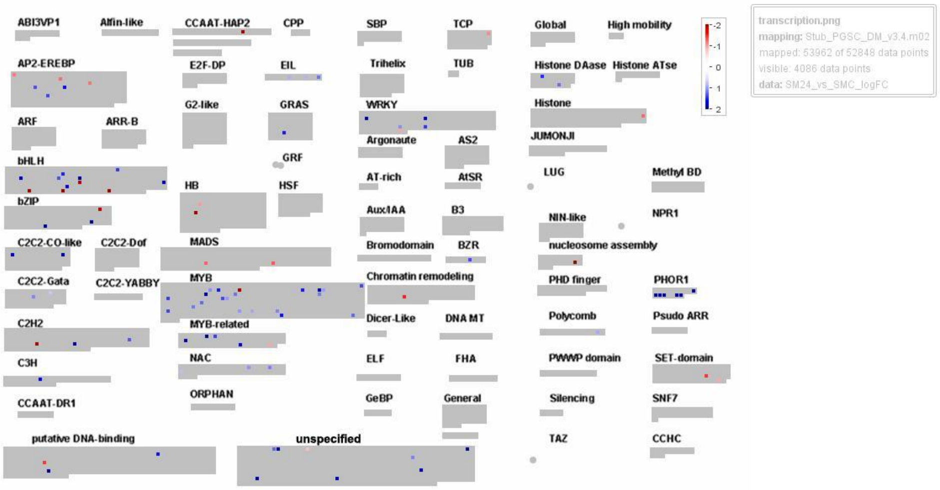

72 h

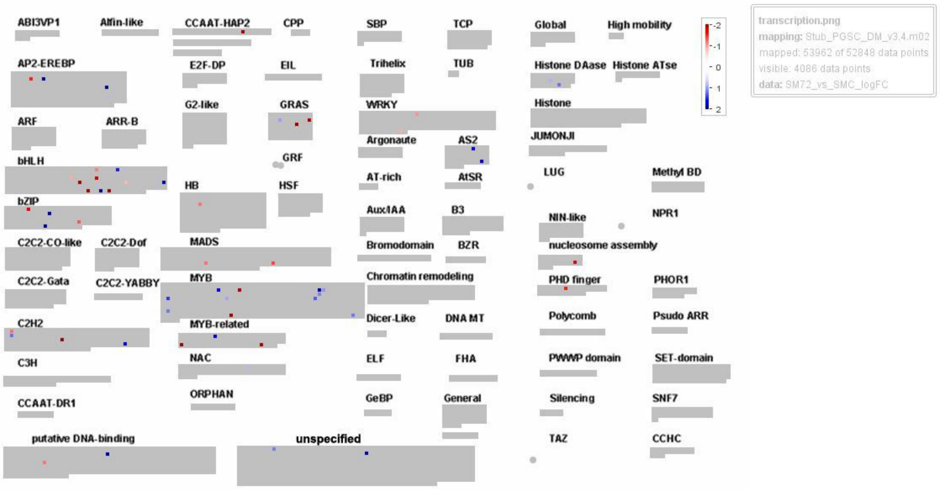

SW-1015 – transciption factors

6 h

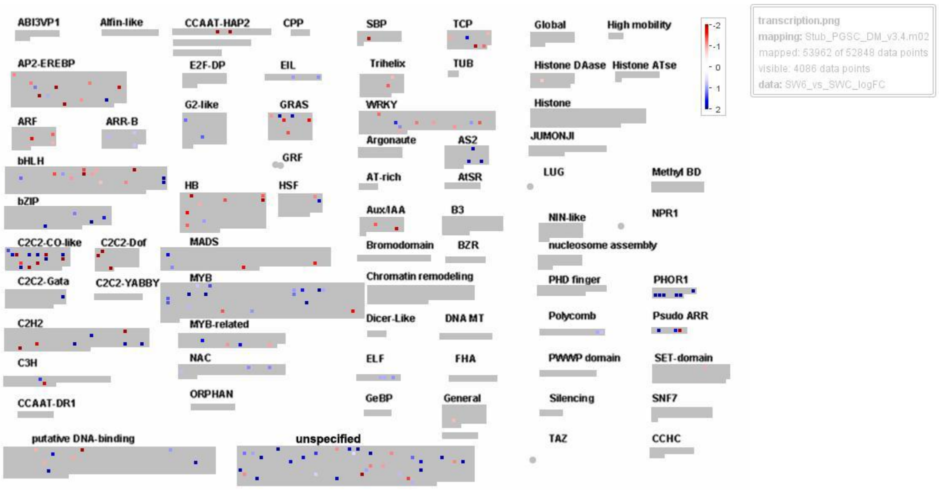

24 h

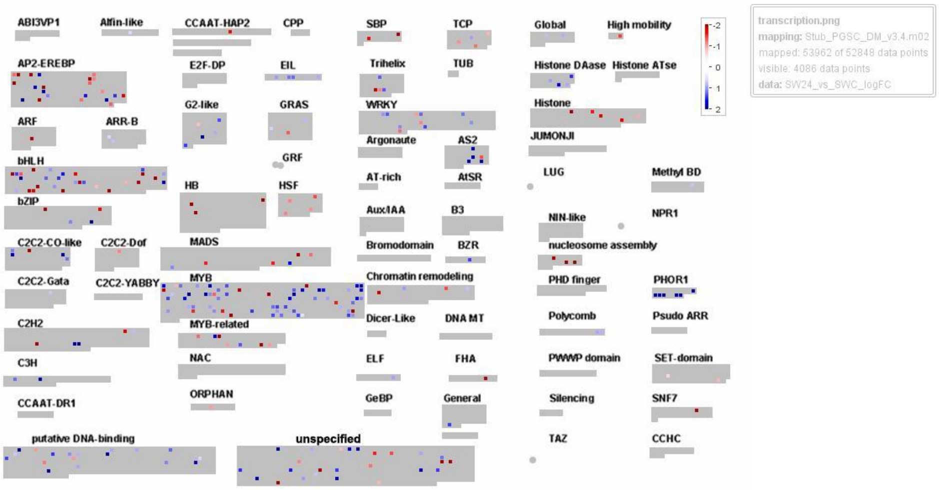

72 h

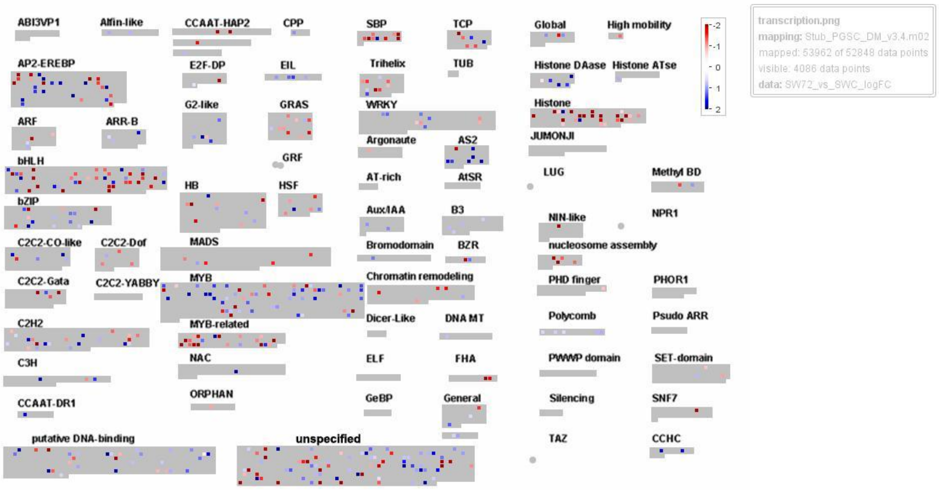

Figure S2b

Desiree – hormones and signalling

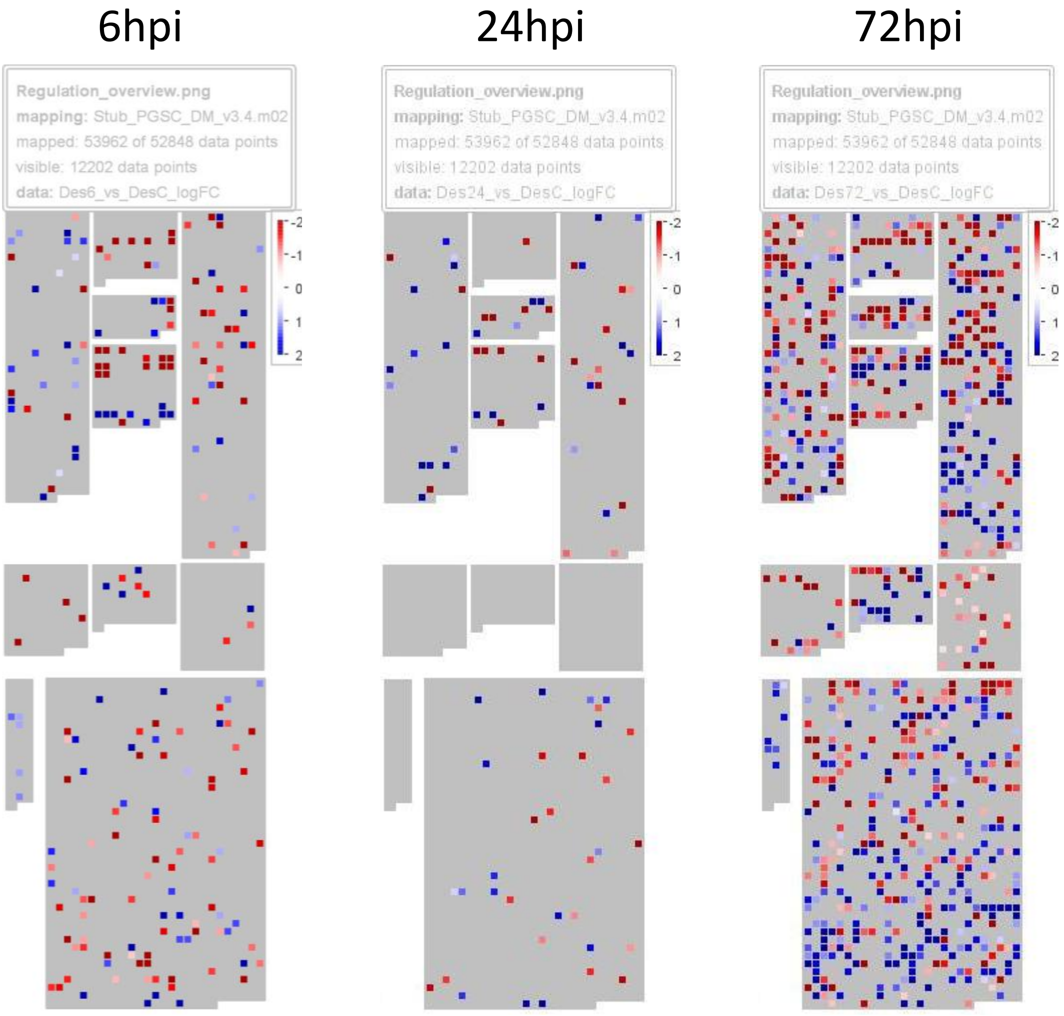

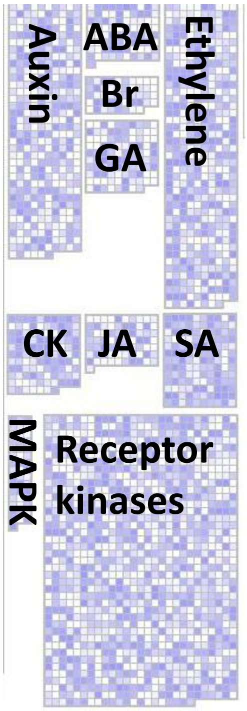

ABA, abscisic acid
BR, brassinosteroids
GA, giberellic acid
CK, cytokinin
JA, jasmonic acid
SA, salicylic acid
MAPK, Mitogen-Activated Protein Kinase

Sarpo Mira – hormones and signalling

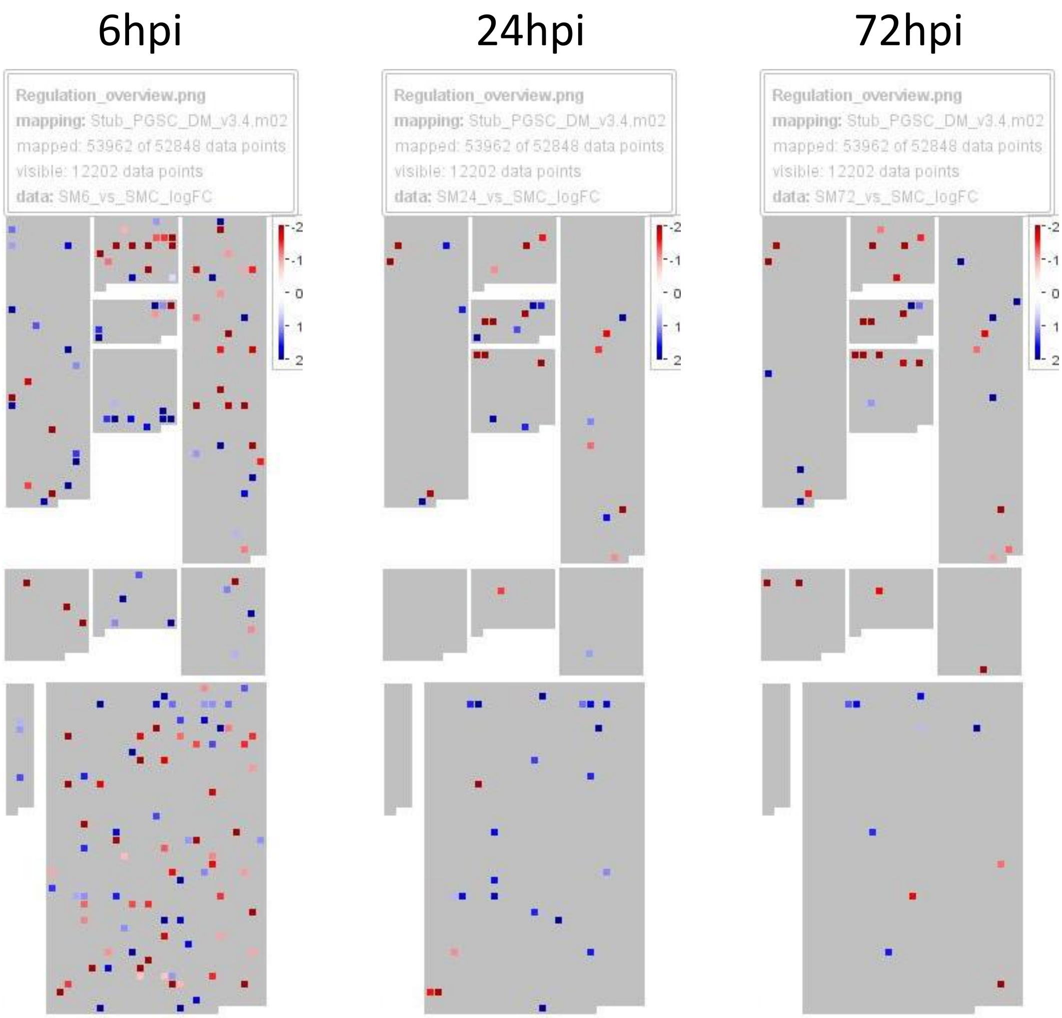

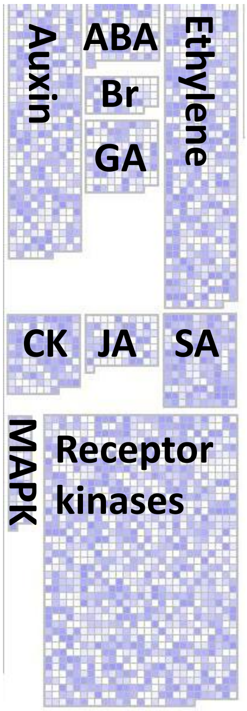

ABA, abscisic acid
BR, brassinosteroids
GA, giberellic acid
CK, cytokinin
JA, jasmonic acid
SA, salicylic acid
MAPK, Mitogen-Activated Protein Kinase
