# Supplementary material for: Quantitative proteomics and transcriptomics of potato in response to Phytophthora infestans in compatible and incompatible interactions
Source: BMC Genomics. 2014 Jun 19;15(1):497. doi: 10.1186/1471-2164-15-497 (PMC4079953; doi:10.1186/1471-2164-15-497)
Supplement: Supplementary file 5 — Additional file 5: Table S4: Protein identifiers for resistance candidates identified in STEM profiles. Excel file with identifiers correspond to profiles presented in Figure 6b-d. (PDF 55 KB) [file 12864_2014_6185_MOESM5_ESM.pdf]

Table S4 Protein identifiers for resistance candidates identified in STEM profiles

Protein ids Fig 6b
PGSC0003DMP400008749
PGSC0003DMP400023388
PGSC0003DMP400030376
PGSC0003DMP400003983
PGSC0003DMP400046980
Q9XIB2
PGSC0003DMP400016669
PGSC0003DMP400003176
PGSC0003DMP400027201
PGSC0003DMP400031346
PGSC0003DMP400031806
PGSC0003DMP400041612
PGSC0003DMP400046178
PGSC0003DMP400051176

Protein ids Fig 6c
F4K409
PGSC0003DMP400004610
PGSC0003DMP400009220
Q42572
Q9LVB8
Q9SKL6
PGSC0003DMP400012829
PGSC0003DMP400038531
PGSC0003DMP400049899
PGSC0003DMP400063086
Q9C876
PGSC0003DMP400017481
F4IB63
PGSC0003DMP400003188
PGSC0003DMP400031840
PGSC0003DMP400044898
PGSC0003DMP400048975
P93814
PGSC0003DMP400007585
PGSC0003DMP400016965
PGSC0003DMP400023730
PGSC0003DMP400026983
PGSC0003DMP400027005
PGSC0003DMP400044902
PGSC0003DMP400045361
PGSC0003DMP400054598
Q9C895
Q9SPM5

Protein ids Fig 6d
F4HRS0
PGSC0003DMP400062387

PGSC0003DMP400048151
PGSC0003DMP400024741
Q9FZB1
PGSC0003DMP400007260
PGSC0003DMP400012143
PGSC0003DMP400043899
