# Supplementary material for: Quantitative proteomics and transcriptomics of potato in response to Phytophthora infestans in compatible and incompatible interactions
Source: BMC Genomics. 2014 Jun 19;15(1):497. doi: 10.1186/1471-2164-15-497 (PMC4079953; doi:10.1186/1471-2164-15-497)
Supplement: Supplementary file 7 — Additional file 7: Table S6: LC-MS file names. Raw data was have been deposited to the ProteomeXchange Consortium (http://proteomecentral.proteomexchange.org) via the PRIDE partner repository with the dataset identifier PXD000435 and DOI 10.6019/PXD000435. (PDF 32 KB) [file 12864_2014_6185_MOESM7_ESM.pdf]

Secretome LC-MS data files

File Name	Uniform Resource Identifier	Size (bytes)	MD5
111202_FL_A10_01.mzML.gz	http://srm.swegrid.se/snic/bils/lu_proteomics/orbi/mzml/201112/111202_FL_A10_01.mzML.gz	1136045993	ff7fdb261742aaa3ac7fc2c63695d5db
111202_FL_A11_01.mzML.gz	http://srm.swegrid.se/snic/bils/lu_proteomics/orbi/mzml/201112/111202_FL_A11_01.mzML.gz	1176849774	240ed13ab75ea17b2b639c048e67f0dc
111202_FL_A12_01.mzML.gz	http://srm.swegrid.se/snic/bils/lu_proteomics/orbi/mzml/201112/111202_FL_A12_01.mzML.gz	1190350642	7219e85eae74d8ec400ad0fe572434f8
111202_FL_A5_01.mzML.gz	http://srm.swegrid.se/snic/bils/lu_proteomics/orbi/mzml/201112/111202_FL_A5_01.mzML.gz	1215666535	0993c75eada6444dcc60dc7b9feaa5b6
111202_FL_A6_01.mzML.gz	http://srm.swegrid.se/snic/bils/lu_proteomics/orbi/mzml/201112/111202_FL_A6_01.mzML.gz	1099872541	b23d7f037f88636635cca7f10d99e0b3
111202_FL_A7_01.mzML.gz	http://srm.swegrid.se/snic/bils/lu_proteomics/orbi/mzml/201112/111202_FL_A7_01.mzML.gz	1131164127	b11427a2cbcd0667e9bc22f689d7c68a
111202_FL_A8_01.mzML.gz	http://srm.swegrid.se/snic/bils/lu_proteomics/orbi/mzml/201112/111202_FL_A8_01.mzML.gz	1158489491	543038be2cf0159fdb999acec034dc3c
111202_FL_A9_01.mzML.gz	http://srm.swegrid.se/snic/bils/lu_proteomics/orbi/mzml/201112/111202_FL_A9_01.mzML.gz	1207851722	b6950614193f9f0f4282f31432bbf017
111202_FL_B1_01.mzML.gz	http://srm.swegrid.se/snic/bils/lu_proteomics/orbi/mzml/201112/111202_FL_B1_01.mzML.gz	1156024957	5ecc8c6d6f7b077232f2f7be304f9ada
111202_FL_B2_01.mzML.gz	http://srm.swegrid.se/snic/bils/lu_proteomics/orbi/mzml/201112/111202_FL_B2_01.mzML.gz	1135104343	e621fe45434a920aa687c8ff86863016
111202_FL_B3_01.mzML.gz	http://srm.swegrid.se/snic/bils/lu_proteomics/orbi/mzml/201112/111202_FL_B3_01.mzML.gz	1084303452	77b5dd8f28e031fceac5804ab73cf56b
111202_FL_B4_01.mzML.gz	http://srm.swegrid.se/snic/bils/lu_proteomics/orbi/mzml/201112/111202_FL_B4_01.mzML.gz	1135425198	04f6a23e34c0d27babda6644f2198435
111202_FL_B5_01.mzML.gz	http://srm.swegrid.se/snic/bils/lu_proteomics/orbi/mzml/201112/111202_FL_B5_01.mzML.gz	1256088838	827e181ba42b0ad12d6f37e1ee0a3402
111202_FL_B6_01.mzML.gz	http://srm.swegrid.se/snic/bils/lu_proteomics/orbi/mzml/201112/111202_FL_B6_01.mzML.gz	1190169295	e3b5be647a2320297a88939e9e12936b
111202_FL_B7_01.mzML.gz	http://srm.swegrid.se/snic/bils/lu_proteomics/orbi/mzml/201112/111202_FL_B7_01.mzML.gz	1100431703	9574a9197f0e430593d5f399de7a586f
111205_FL_A1_02.mzML.gz	http://srm.swegrid.se/snic/bils/lu_proteomics/orbi/mzml/201112/111205_FL_A1_02.mzML.gz	1050715476	93f34726b7f4a0972f1e5338346f6dd3
111205_FL_A2_02.mzML.gz	http://srm.swegrid.se/snic/bils/lu_proteomics/orbi/mzml/201112/111205_FL_A2_02.mzML.gz	1096188626	4c8114525f078554650894c3baf521f4
111205_FL_A3_02.mzML.gz	http://srm.swegrid.se/snic/bils/lu_proteomics/orbi/mzml/201112/111205_FL_A3_02.mzML.gz	1108565762	5f099af4562a3a822405dc3daf8a54d3
111205_FL_A4_02.mzML.gz	http://srm.swegrid.se/snic/bils/lu_proteomics/orbi/mzml/201112/111205_FL_A4_02.mzML.gz	1007839691	62f62ef8b9d5bcdd440ce70770ae1d5f
111205_FL_C10_01.mzML.gz	http://srm.swegrid.se/snic/bils/lu_proteomics/orbi/mzml/201112/111205_FL_C10_01.mzML.gz	1064542814	7530ae4dd6e565d9de95bafd1325164c
111205_FL_C12_01.mzML.gz	http://srm.swegrid.se/snic/bils/lu_proteomics/orbi/mzml/201112/111205_FL_C12_01.mzML.gz	1055941582	482679d59200fda908919988641361b7
111205_FL_C6_01.mzML.gz	http://srm.swegrid.se/snic/bils/lu_proteomics/orbi/mzml/201112/111205_FL_C6_01.mzML.gz	1038992380	661cc47e1d9ec8c53d0e211cf46b4de7
111206_FL_B10_01.mzML.gz	http://srm.swegrid.se/snic/bils/lu_proteomics/orbi/mzml/201112/111206_FL_B10_01.mzML.gz	1141389566	977c4367b7e8f153c3f30a2f6863587b
111206_FL_B11_01.mzML.gz	http://srm.swegrid.se/snic/bils/lu_proteomics/orbi/mzml/201112/111206_FL_B11_01.mzML.gz	1150192251	e47eb6462fd5252afa0509360530eb7f
111206_FL_B12_01.mzML.gz	http://srm.swegrid.se/snic/bils/lu_proteomics/orbi/mzml/201112/111206_FL_B12_01.mzML.gz	1178063735	669d145e9d979dc666a8e6572feecfc4
111206_FL_B8_01.mzML.gz	http://srm.swegrid.se/snic/bils/lu_proteomics/orbi/mzml/201112/111206_FL_B8_01.mzML.gz	1175996507	45fc88b1677aeece5a70add811978c23
111206_FL_B9_01.mzML.gz	http://srm.swegrid.se/snic/bils/lu_proteomics/orbi/mzml/201112/111206_FL_B9_01.mzML.gz	1156952624	4ea92bc7295239de558566fe81c7f0ab
111206_FL_C1_01.mzML.gz	http://srm.swegrid.se/snic/bils/lu_proteomics/orbi/mzml/201112/111206_FL_C1_01.mzML.gz	1046270839	ab8bc64b511c2ab22c343b489e8a6d99
111206_FL_C2_01.mzML.gz	http://srm.swegrid.se/snic/bils/lu_proteomics/orbi/mzml/201112/111206_FL_C2_01.mzML.gz	1027252391	c2e936a2a1e5771cd95360abede21023
111207_FL_C11_01.mzML.gz	http://srm.swegrid.se/snic/bils/lu_proteomics/orbi/mzml/201112/111207_FL_C11_01.mzML.gz	1087051082	ef23bdb53ef8925908f5196b8f8b3e96
111207_FL_C3_01.mzML.gz	http://srm.swegrid.se/snic/bils/lu_proteomics/orbi/mzml/201112/111207_FL_C3_01.mzML.gz	1130054874	cf71351efa23a58bde928a7324015b38
111207_FL_C4_01.mzML.gz	http://srm.swegrid.se/snic/bils/lu_proteomics/orbi/mzml/201112/111207_FL_C4_01.mzML.gz	1072961898	f1b4e259aba0fc6ab5832b9ff466e4be
111207_FL_C5_01.mzML.gz	http://srm.swegrid.se/snic/bils/lu_proteomics/orbi/mzml/201112/111207_FL_C5_01.mzML.gz	1166818097	5a4f1f907d587a7e7b23c08ee6fdcae5
111207_FL_C7_01.mzML.gz	http://srm.swegrid.se/snic/bils/lu_proteomics/orbi/mzml/201112/111207_FL_C7_01.mzML.gz	1098169221	6a593ba1ab7fa8a18a187ac47c655d93
111207_FL_C8_01.mzML.gz	http://srm.swegrid.se/snic/bils/lu_proteomics/orbi/mzml/201112/111207_FL_C8_01.mzML.gz	1055214463	21c9b0f7a1d60e35923f74f1a68ab6af
111207_FL_C9_01.mzML.gz	http://srm.swegrid.se/snic/bils/lu_proteomics/orbi/mzml/201112/111207_FL_C9_01.mzML.gz	992586521	b33c58342c184985d3f023ac3aae6d13
120427_FL_A13_01.mzML.gz	http://srm.swegrid.se/snic/bils/lu_proteomics/orbi/mzml/201204/120427_FL_A13_01.mzML.gz	1116882353	f8ab06e611d7000b5c6556fd3a2d87d3
120427_FL_A14_01.mzML.gz	http://srm.swegrid.se/snic/bils/lu_proteomics/orbi/mzml/201204/120427_FL_A14_01.mzML.gz	1121152452	c6f20ba86a68bea1822b0dbe34919d02
120427_FL_A15_01.mzML.gz	http://srm.swegrid.se/snic/bils/lu_proteomics/orbi/mzml/201204/120427_FL_A15_01.mzML.gz	1068253382	59118b169bf4ebe361a7c445900cd81d
120427_FL_A16_01.mzML.gz	http://srm.swegrid.se/snic/bils/lu_proteomics/orbi/mzml/201204/120427_FL_A16_01.mzML.gz	974874313	638a0c6656dffc9e3a6efadf527b00c6
120427_FL_A17_01.mzML.gz	http://srm.swegrid.se/snic/bils/lu_proteomics/orbi/mzml/201204/120427_FL_A17_01.mzML.gz	1011353135	188f05521de46e251a517f77cc0e6303
120427_FL_A18_01.mzML.gz	http://srm.swegrid.se/snic/bils/lu_proteomics/orbi/mzml/201204/120427_FL_A18_01.mzML.gz	1008258550	de77a36151ad813cb51bb404acd1b805
120427_FL_A19_01.mzML.gz	http://srm.swegrid.se/snic/bils/lu_proteomics/orbi/mzml/201204/120427_FL_A19_01.mzML.gz	1047571367	c43a185245b99824229a9462f45494e4
120427_FL_A20_01.mzML.gz	http://srm.swegrid.se/snic/bils/lu_proteomics/orbi/mzml/201204/120427_FL_A20_01.mzML.gz	981146661	805214b108fac307f99aa110313220d1
120427_FL_B13_01.mzML.gz	http://srm.swegrid.se/snic/bils/lu_proteomics/orbi/mzml/201204/120427_FL_B13_01.mzML.gz	1090576857	26c8d227c199d68b1cd7842a0f323a7b
120427_FL_B14_01.mzML.gz	http://srm.swegrid.se/snic/bils/lu_proteomics/orbi/mzml/201204/120427_FL_B14_01.mzML.gz	1144565982	21dbb452c5c9baad6fbd584f298488d0
120427_FL_B15_01.mzML.gz	http://srm.swegrid.se/snic/bils/lu_proteomics/orbi/mzml/201204/120427_FL_B15_01.mzML.gz	1049283906	2e95022cff6d689d985773f1e9ec9e72
120427_FL_B16_01.mzML.gz	http://srm.swegrid.se/snic/bils/lu_proteomics/orbi/mzml/201204/120427_FL_B16_01.mzML.gz	999432811	4888333e12d8316376c1718d803f0712
120427_FL_B17_01.mzML.gz	http://srm.swegrid.se/snic/bils/lu_proteomics/orbi/mzml/201204/120427_FL_B17_01.mzML.gz	1008804559	c65821b937dc6c6a8f2aa822da0c6b14
120427_FL_B18_01.mzML.gz	http://srm.swegrid.se/snic/bils/lu_proteomics/orbi/mzml/201204/120427_FL_B18_01.mzML.gz	1059384358	e3a777e1d22f09884a4954e0d66f253f
120427_FL_B19_01.mzML.gz	http://srm.swegrid.se/snic/bils/lu_proteomics/orbi/mzml/201204/120427_FL_B19_01.mzML.gz	1023420253	0ec6721bd94ddc939b00a53f65a0601c
120427_FL_B20_01.mzML.gz	http://srm.swegrid.se/snic/bils/lu_proteomics/orbi/mzml/201204/120427_FL_B20_01.mzML.gz	957751314	b8f80d031601f777e82ff6fcd4402856
120427_FL_C13_01.mzML.gz	http://srm.swegrid.se/snic/bils/lu_proteomics/orbi/mzml/201204/120427_FL_C13_01.mzML.gz	1073720716	9d1fdf5991f3020b3b552f3fa9719e99
120427_FL_C14_01.mzML.gz	http://srm.swegrid.se/snic/bils/lu_proteomics/orbi/mzml/201204/120427_FL_C14_01.mzML.gz	1079872629	80b6326494a411be386d06841c22a0ff
120427_FL_C15_01.mzML.gz	http://srm.swegrid.se/snic/bils/lu_proteomics/orbi/mzml/201204/120427_FL_C15_01.mzML.gz	997739642	a052ee403c7473f010c26006ff22147d
120427_FL_C16_01.mzML.gz	http://srm.swegrid.se/snic/bils/lu_proteomics/orbi/mzml/201204/120427_FL_C16_01.mzML.gz	982607852	d717e6b9ff27a8050c0254bf7c5c3449
120427_FL_C17_01.mzML.gz	http://srm.swegrid.se/snic/bils/lu_proteomics/orbi/mzml/201204/120427_FL_C17_01.mzML.gz	979996961	5bf0bb55690daa3c41d9e7ef2b18aa28
120427_FL_C18_01.mzML.gz	http://srm.swegrid.se/snic/bils/lu_proteomics/orbi/mzml/201204/120427_FL_C18_01.mzML.gz	1028012622	f5483b0d09373c03889530ad38dd2bbf
120427_FL_C19_01.mzML.gz	http://srm.swegrid.se/snic/bils/lu_proteomics/orbi/mzml/201204/120427_FL_C19_01.mzML.gz	1042220055	003ee02cfc60a54349206691904dac62
120427_FL_C20_01.mzML.gz	http://srm.swegrid.se/snic/bils/lu_proteomics/orbi/mzml/201204/120427_FL_C20_01.mzML.gz	1022715759	1828f1e5d4fe346d52a00ed193f3d9b6
